# DPP4 Deficiency Preserved Cardiac Function in Abdominal Aortic Banding Rats

**DOI:** 10.1371/journal.pone.0085634

**Published:** 2014-01-09

**Authors:** Hui-Chun Ku, Ming-Jai Su

**Affiliations:** Institute of Pharmacology, College of Medicine, National Taiwan University, Taipei, Taiwan; Second University of Naples, Italy

## Abstract

Dipeptidyl peptidase-4 (DPP4) enzyme inhibition has been reported to increase plasma glucagon-like peptide-1 (GLP-1) level for controlling postprandial glucose concentration. A prominent GLP-1 level in DPP4-deficient rats contributed to the resistance of endotoxemia and myocardial infarction. DPP4 deficiency also increased the capability against H_2_O_2_-induced stress in cardiomyocyte. However, long term effect of loss DPP4 activity on cardiac performance remained unclear. We used abdominal aortic banding (AAB) to induce pressure overload in wild-type and DPP4-deficient rats, and investigated the progression of heart failure. Cardiac histology and function were determined. Blood sample was collected for the plasma biochemical marker measurement. Heart weight to body weight ratio increased 1.2-fold after 6 weeks of AAB surgery. Cardiac function was compensated against pressure overload after 6 weeks of AAB surgery, but progressed to deterioration after 10 weeks of AAB surgery. AAB induced cardiac dysfunction was alleviated in DPP4-deficient rats. DPP4 activity increased significantly in wild-type rats after 10 weeks of AAB surgery, but remained unchanged in DPP4-deficient rats. In contrast, GLP-1 concentration was elevated by AAB after 6 weeks of surgery in DPP4-deficient rats, and remained high after 10 weeks of surgery. Ang II level markedly increased after 6 weeks of AAB surgery, but were less in DPP4-deficient rats. Massive collagen deposits in wild-type rat hearts appeared after 10 weeks of AAB surgery, which were alleviated in DPP4-deficient rats. Long term deficiency of DPP4 activity improved cardiac performance against pressure overload in rat, which may be attributed to a great quantity of GLP-1 accumulation during AAB.

## Introduction

Heart failure remains a leading cause of death in patients. Sustained pressure overload stimulates pathological cardiac hypertrophy, which may lead to heart failure [1]. Insulin resistance has been reported to occur with increased left ventricular mass and cardiac hypertrophy [2], and also lead to high incidence of heart failure [2], [3]. Diabetes impairs insulin sensitivity which results in a shift of myocardial substrate usage towards even higher fatty acid utilization [4], which contributes to myocardial dysfunction [5]. Changes in myocardial structure, function and metabolism have been described in diabetes, which link to high risk factor of cardiovascular disease, so the medication that decreases cardiovascular risk factor in the diabetic subject is an important issue. However, the efficacy of current therapy to prevent cardiovascular disease in diabetes is still limited.

Incretin hormones have recently been developed as a new target for the maintenance of systemic glucose homeostasis. Glucagon-like peptide-1 (GLP-1), one of the incretin hormones, is secreted from the gastrointestinal tract after food intake and stimulates glucose-dependent insulin secretion by activating GLP-1 receptor [6]. When cleaved by dipeptidyl peptidase-4 (DPP4), GLP-1 is characterized by a complete lack of insulinotropic activity [7]. DPP4 is an enzyme that catalyzes the release of dipeptides from the N terminus which contain Pro or Ala in the third amino acid position [7]. GLP-1-based therapies have been approved recently as diabetes treatment, such as GLP-1 analogue and DPP4 inhibitor [8]–[10]. In addition, GLP-1 receptor is expressed in the cardiovascular system [11], [12], and modulates cardiac function. GLP-1 signaling has been demonstrated to improve cardiac function in several animal model experiments, such as endotoxemia, myocardial infarction, and diabetic cardiomyopathy [11], [13]–[18]. All these experiments evaluate that the cytoprotective effect of GLP-1 is mediated mainly by the activation of survival pathway, inhibition of apoptosis pathway, and improvement of glucose utilization to against oxidative and inflammatory stress in heart.

Accumulating studies have indicated that DPP4 inhibitors also play an important role on cardiovascular system. Lack of DPP4 activity acts to decrease myocardial infarct size, stabilize the cardiac electrophysiological state during myocardial ischemia, reduce ischemia/reperfusion injury, and prevent left ventricular remodeling after myocardial infarction [14], [15], [19], [20]. In the cell study, DPP4 deficiency exerts protective effect against H_2_O_2_-induced stress in isolated cardiomyocytes [21]. Long term deficiency of DPP4 induces changes of cellular function, and increase the capability against oxidative stress via enhancing AKT signaling and glucose uptake in association with preserving catalase activity, via both GLP-1 receptor dependent and independent signaling [21]. Moreover, DPP4 inhibitors also protect the vasculature through their anti-inflammatory, anti-atherosclerotic effects, and promote vascular relaxation. [19]. Several clinical studies were also performed to investigate the safety of DPP4 inhibitors on diabetes with cardiovascular disease. DPP4 inhibitors improve several risk factors in patients with T2DM, and the positive effects on heart have also been described in patients with ischemic heart disease or congestive heart failure [10], [22], [23]. However, a controversy study was also found. Saxagliptin did not affect the rate of ischemic events, but increased the rate of hospitalization for heart failure [24]. The potential explanation for the differential studies is the exposure time of the drug may not long enough and the difference of glycated hemoglobin levels between patients [24].

Accordingly, long term safety of DPP4 inhibitor remains unclear. Abdominal aortic banding is one of the most widely used animal models in induction of heart failure [25]. As the animal grows, the aortic outflow is increasingly impeded, and hypertension is developed gradually. In this context, we aimed to investigate the progression of cardiac response after pressure overload surgery in both wild-type and DPP4-deficient rats.

## Materials and Methods

### 1. Experimental Animals

The research was performed in accordance with the Guide for the Care and Use of Laboratory Animals published by the US National Institutes of Health (NIH publication no. 85–23, revised 1996), and was approved by the Institutional Animal Care and Use Committee of the National Taiwan University. Adult male Fischer 344 and DPP4-deficient mutant (DchcHsd-DPP IV) rats were purchased from National Laboratory Animal Center of Taiwan and Harlan laboratories, Inc, respectively. Fischer 344 is the control strain for the DPP4-deficient rats. Rats were maintained under a 12-h light/dark cycle at a controlled temperature (21±2°C) with free access to food and tap water.

### 2. Experimental Model of Pressure-overload in vivo

Pressure-overload was induced by abdominal aortic banding (AAB) as described previously [26]. Briefly, rats (8 week old) were anesthetized with pentobarbital (60 mg/kg, i.p.). After rats appeared calm, the abdominal aorta was isolated adjacent to renal arteries, and constricted by a 4–0 silk suture ligature tied firmly against a 22-gauge needle. The needle was removed immediately and performed a constriction of 0.7 mm in diameter. Sham-operated rat underwent a similar surgical procedure without aortic constriction. After operation, rats were placed on an electric blanket to maintain body temperature until awake, and monitor after 6 or 10 weeks of surgery.

### 3. Determination of Cardiac Function in vivo

Cardiac function was estimated in vivo by a pressure-volume catheter (1.9 F; Scisense Instruments, Ontario, Canada) as described previously [14], [15], [27]. After 6 or 10 weeks of surgery, rats were anesthetized, and the right carotid artery was cannulated with a catheter, which connected to a control box with signal conditioning and amplification circuitry (112B-C009, Scisense, Canada). Then the catheter was advanced into the left ventricle for the measurement of pressure and volume. PV loops were recorded in basal condition, and several parameters were analyzed. PV loops were then measured under the condition of preload change, which induced by inferior vena cava constriction as described previously [14], [15], [27]. The end-systolic pressure-volume relation (ESPVR) and end-diastolic pressure-volume relation (EDPVR) are characterized by the slope of end-systolic and end-diastolic pressure-volume point, which are load-independent indexes of myocardial contractility and ventricular compliance, respectively.

### 4. Determination of Cardiac Histology

After the hemodynamic data recordings, the heart was rapidly removed and perfused on a modified Langendorff perfusion apparatus with Krebs buffer (containing: 110 mM NaCl, 2.6 mM KCl, 1.2 mM KH_2_PO_4_, 1.2 mM MgSO_4_, 25 mM NaHCO_3_ and 11 mM glucose (pH 7.4)). After blood was washed out, the weight of heart was measured, and calculated the heart to body weight ratio (HW/BW). The hearts were fixed in 4% paraformaldehyde, embedded in paraffin, and sectioned horizontally in 4 µm slice. HE and masson’s trichrome stain were performed for histological and fibrosis analysis.

### 5. Determination of Plasma DPP4 Activity, GLP-1, and Angiotensin II Concentration

Blood sample was collected and centrifuged at 13,000 rpm for 5 min, and plasma was stored at −80°C until used. Gly-Pro-AMC (St. Louis, MO, USA), a synthesis substrate of DPP4, was used for the determination of DPP4 activity as described previously. The amount of AMC liberation is positive related with DPP4 activity. Plasma in 50 mM HEPES solution containing 100 uM Gly-Pro-AMC was incubated at 37°C for 60 min, and the liberation of free AMC was monitored at 370-nm excitation and 440-nm emission. Plasma GLP-1 levels were measured by ELISA kit (Millipore Corporation, Billerica, MA, USA). Plasma angiotensin II levels were measured by ELISA kit (BioRay, Laguna Hills, CA, USA).

### 6. Statistical Analysis

All values were presented as means ± SE. The results were analyzed using ANOVA followed by Bonferroni’s post hoc tests. P<0.05 was considered as significant difference.

## Results

### 1. AAB-induced Cardiac Dysfunction was Recovered in DPP-4 Deficient Rats

**Figure 1:**
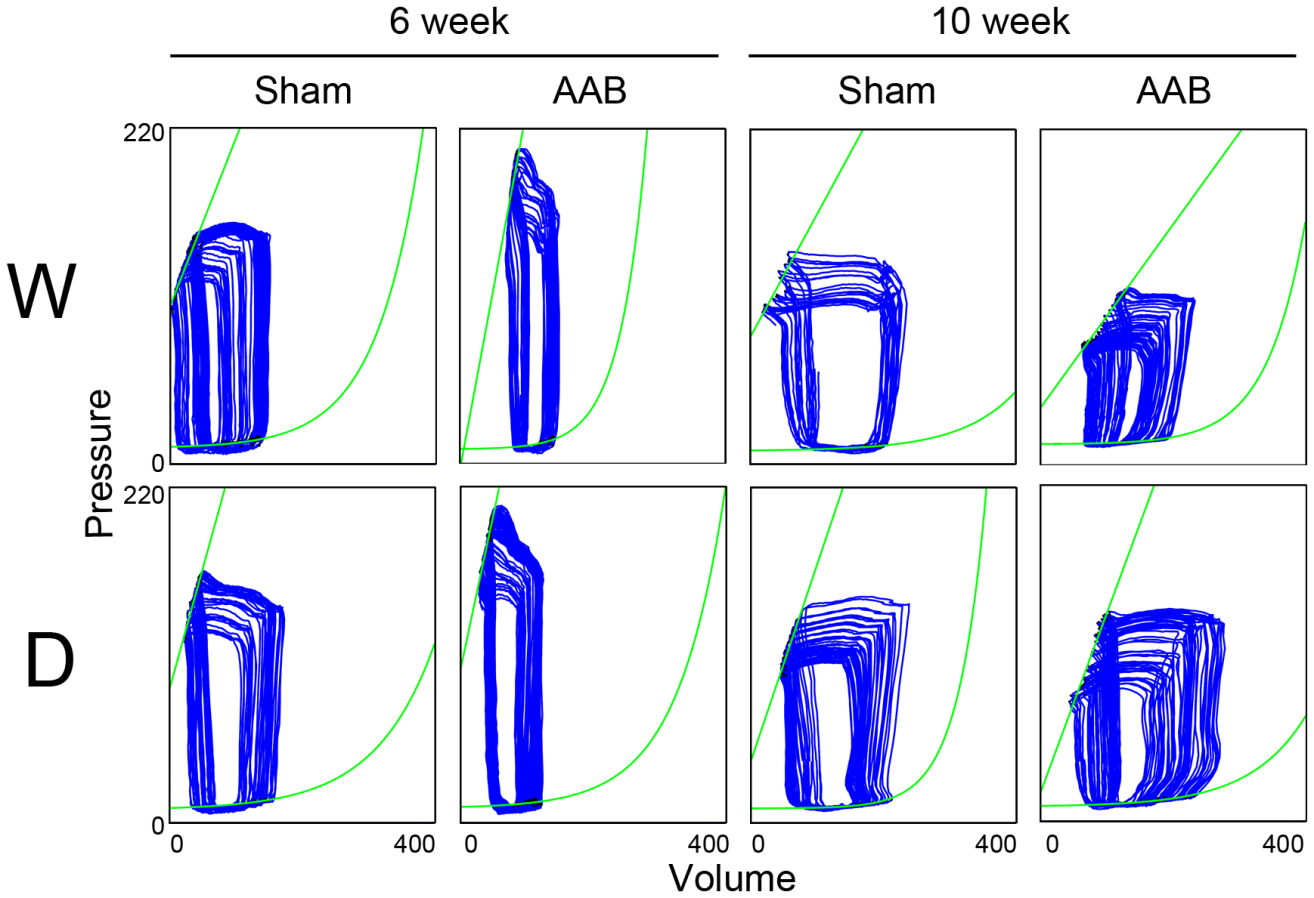
Pressure-volume loops of wild-type and DPP4-deficient rat hearts in sham and AAB conditions after 6 or 10 weeks of surgery. W indicates wild-type, and D indicates DPP4 deficiency.

The cardiac functions were measured by PV loops in the sham and AAB conditions after 6 and 10 weeks of surgery. The representative PV loops were shown (Fig. 1), and the hemodynamic data were all quantified (Table 1). In the 6-week sham condition, similar cardiac performance was found in two kinds of rats. After 6 weeks of AAB surgery, cardiac function was compensated against pressure overload. AAB caused an enhancement of LVSP in wild-type rats, being 1.17-fold of sham. LVESV also increased after AAB surgery, being 2.2-fold of sham, while LVEDV remained unchanged, so both SV and EF were reduced in wild-type rats. The compensatory cardiac response to AAB was also found in DPP4-deficient rats. LVSP was elevated to 1.21 fold of sham in DPP4-deficient rats after 6 weeks of AAB. However, the increase of LVESV was less in DPP4-deficient rats; therefore, SV and EF remained. ESPVR and EDPVR, load-independent cardiac function indexes, elevated more after AAB in wild-type rats, suggesting that AAB resulted in the increase of myocardial contractility and stiffness, whereas EDPVR in DPP4-deficient rats remained unchanged after 6 week of AAB.

**Table 1:** Invasive hemodynamic analysis of 6 and 10 weeks after AAB in wild-type and DPP4-deficient rats.

	Wild-type		DPP4-deficiency	
	Sham	AAB	Sham	AAB
6 week				
LVSP (mmHg)	147.9±6.0	172.8±4.9*	153.9±2.9	185.8±6.5†
LVEDP (mmHg)	4.5±0.3	5.6±0.5	4.9±0.5	5.3±1.0
LVDP (mmHg)	143.4±5.8	167.2±5.2*	148.3±3.2	180.5±7.5†
HR (bpm)	396.1±10.8	389.9±21.5	405.4±8.5	395.4±24.0
+max dPdt (mmHg/s)	14243.5±1069.4	13778.4±695.6	14445.6±114.6	13974.1±253.7
−min dPdt (mmHg/s)	12700.2±1129.1	12229.8±753.9	12525.8±193.9	12539.9±168.9
LVESV (µL)	36.2±5.7	81.4±15.9*	36.2±2.0	43.3±9.5#
LVEDV (µL)	143.7±7.2	161.1±7.7	155.9±18.8	141.8±3.4
SV (µL)	107.5±3.1	79.7±11.9*	119.7±17.6	98.4±7.9#
%EF	75.3±2.9	50.5±10.7*	75.9±1.9	69.3±7.4#
ESPVR slope (mmHg/µL)	1.21±0.05	2.30±0.45*	1.31±0.11	1.97±0.05†
EDPVR slope (mmHg/µL)	0.0220±0.0026	0.0416±0.0089*	0.0230±0.0031	0.0194±0.0083
10 week				
LVSP (mmHg)	136.2±7.2	119.6±8.9^@^	141.7±3.5	140.3±7.2&
LVEDP (mmHg)	4.7±0.5	6.8±1.9^@^	4.5±0.1	4.4±0.9&
LVDP (mmHg)	131.4±7.1	112.8±8.1^@^	137.2±4.2	135.9±8.1&
HR (bpm)	378.1±35.0	366.3±13.3	393.7±15.2	385.3±17. 3
+max dPdt (mmHg/s)	11353.8±652.5	8568.2±483.9^@^	10924.9±945.5	10039.7±246.4&
−min dPdt (mmHg/s)	8301.3±683.9*	6994.9±519.9^@^	10696.8±1028.7	8644.8±850.2&
LVESV (µL)	64.6±11.4	125.3±2.8^@^	67.5±7.9	89.2±14.7&
LVEDV (µL)	194.9±26.7	231.1±19.7	213.2±38.7	249.2±37.3
SV (µL)	130.2±16.2	105.9±18.1^@^	145.7±31.2	159.9±22.7&
%EF	67.5±2.5	44.7±4.6^@^	67.6±2.2	64.4±0.9&
ESPVR slope (mmHg/µL)	1.10±0.35	0.59±0.20^@^	1.12±0.23	0.82±0.20&
EDPVR slope (mmHg/µL)	0.0145±0.0033	0.0126±0.0023	0.0172±0.0069	0.0168±0.0027

Values are means ± SE (n = 8) MAP : mean arterial pressure, HR : heart rate, LVSP : left ventricular systolic pressure, LVEDP : left ventricular end-diastolic pressure, DP : development pressure, +dP/dt and −dP/dt : maximal slope of the systolic pressure increment and diastolic pressure decrement, respectively, Ves : end-systolic volume, Ved : end-diastolic volume, SV : stroke volume, EF : ejection fraction, ESPVR : end-systolic pressure–volume relation, and EDPVR : end-diastolic pressure–volume relation.

p<0.05 vs. wild-type 6 week sham,

^#^ p<0.05 vs. wild-type 6 week AAB,

^@^ p<0.05 vs. wild-type 10 week sham,

^&^ p<0.05 vs. wild-type 10 week AAB,

p<0.05 vs. DPP4 deficiency 6 week sham.

Without AAB, aging worsen ventricular contractility and relaxation performance, especially the rate of relaxation was suppressed more in wild-type than in DPP4-deficient rats in the 10-week sham condition. Cardiac function was deteriorated after 10 weeks of AAB surgery. In wild-type rats, the reduction of LVSP and the elevation of LVEDP were found, being 0.87- and 1.45-fold of sham group, respectively. Furthermore, the suppression of +dP/dt and –dP/dt were also observed, indicating the deterioration of systolic and diastolic functions. AAB caused a more significant elevation of LVESV in wild-type rats than that in DPP4-deficient rats, being 1.94- and 1.32-fold of sham group, respectively, which resulted in a more prominent reduction of EF in wild-type rats. AAB induced cardiac dysfunction was preserved in DPP4-deficient rats.

### 2. Cardiac Hypertrophy after AAB Surgery

**Figure 2:**
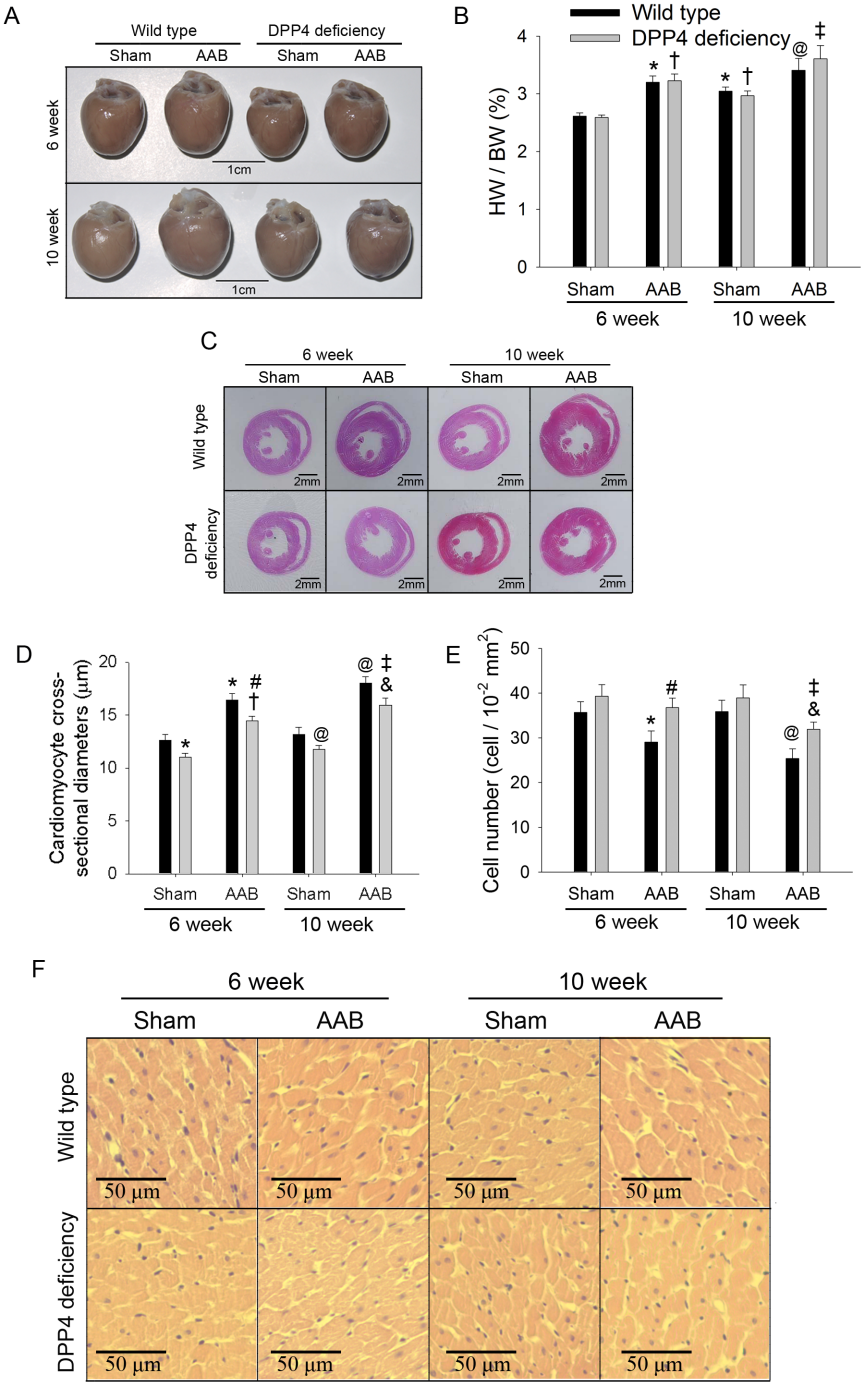
Effects of AAB on heart morphology. Representative heart (A), heart weight to body weight ratio (n = 8) (B), and histological sections (C and F) of HE staining were shown after 6 and 10 weeks of AAB surgery. Cardiomyocyte cross-sectional diameter (D) and cell number (E) were also measured. (300 cell in 5 rats each). *p<0.05 vs. wild-type 6 week sham, ^#^p<0.05 vs. wild-type 6 week AAB, ^@^p<0.05 vs. wild-type 10 week sham, ^&^p<0.05 vs. wild-type 10 week AAB, ^†^p<0.05 vs. DPP4 deficiency 6 week sham, ^‡^p<0.05 vs. DPP4 deficiency 10 week sham.

The heart weight to body weight ratio were measured (Fig. 2B), and the representative heart from each condition were shown (Fig. 2A). DPP4-deficient rats gained weight slower than wild-type rats [28]. Male wild-type rats gained about 40 g weight during 4 weeks, but DPP4-deficient rats gained less than 20 g weight. Heart weight and body weight were both less in DPP4-deficient rats than that in wild-type rats. In the 6-week sham group, HW/BW is similar in two rats. Without AAB, aging can increase HW/BW slightly. After 6 week of AAB, HW/BW increased in wild-type rats, being 1.2-fold of sham, and remained higher after 10 week of AAB. There was no significant difference in HW/BW between two kinds of rats.

Histological analysis of hematoxylin and eosin-stained cardiac sections was shown (Fig. 2C and F). After 10 weeks of AAB surgery, the dimension of left ventricle posterior wall and interventricular septum increased in wild-type rats, along with a smaller ventricular cavity. Cardiomyocyte cross-sectional diameter and cell number were calculated (Fig. 2D and E). In the sham condition, cardiomyocyte was little bigger in wild-type rats than that in DPP4-deficient rats. Morphology analyses showed that AAB induced a marked time-dependent increase in diameter of cardiomyocyte associated with the loss of cell number in wild-type hearts. These changes were less in DDP4-deficient hearts.

### 3. GLP-1 Signaling Upregulated in DPP4-deficient Rats

**Figure 3:**
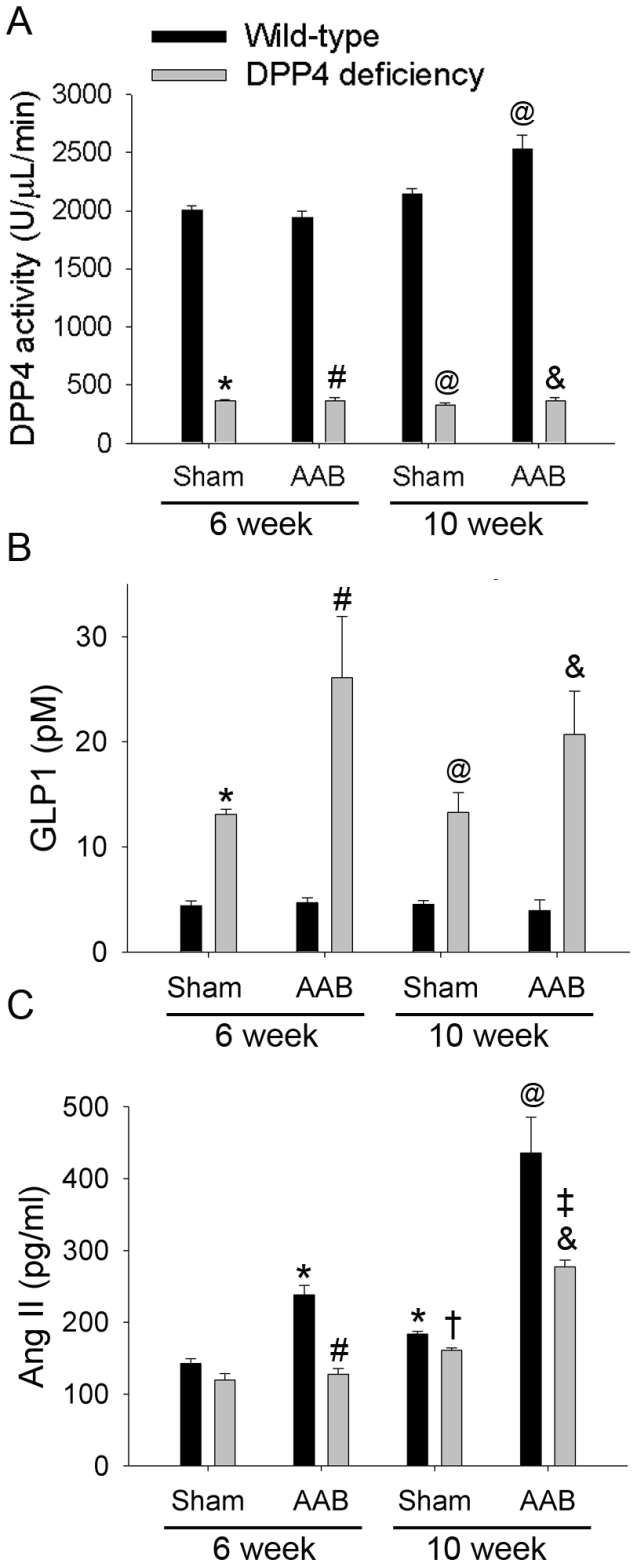
Effects of AAB on plasma GLP-1, DPP4 activity, and Ang II level. Plasma DPP4 activity (A), plasma GLP-1 concentration (B), and plasma Ang II level (C) were measured in wild-type and DPP4-deficient rats after 6 and 10 weeks of AAB surgery. (n = 8 ) *p<0.05 vs. wild-type 6 week sham, ^#^p<0.05 vs. wild-type 6 week AAB, ^@^p<0.05 vs. wild-type 10 week sham, ^&^p<0.05 vs. wild-type 10 week AAB, ^†^p<0.05 vs. DPP4 deficiency 6 week sham, ^‡^p<0.05 vs. DPP4 deficiency 10 week sham.

In the 6-week sham groups, DPP4 activity was 5.5-fold higher in wild-type rats than that in DPP4-deficient rats (Fig. 3A). AAB did not alter DPP4 activity in two kinds of rats after 6 weeks of surgery. However, DPP4 activity increased in wild-type rats after 10 weeks of AAB surgery, but it remained unchanged in DPP4-deficient rats.

In the 6-week sham condition, plasma GLP-1 concentration was 3.0–fold higher in DPP4-deficient rats than that in wild-type rats (Fig. 3B). AAB surgery did not alter GLP-1 level in wild-type rats. However, in DPP4-deficient rats, GLP-1 concentration was elevated by AAB after 6 weeks of surgery, and it remained higher than sham group after10 weeks.

### 4. AAB Induced Ang II Level Elevation was Alleviated in DPP4-deficient Rats

Angiotensin II (Ang II) plays an important role in the progression of pressure overload-induced heart failure. Without AAB, aging cause Ang II concentration increased in both rats (Fig. 3C). Ang II level markedly increased after 6 weeks of AAB surgery, and became much higher after 10 weeks. This elevation was alleviated in DPP4-deficient rats.

### 5. AAB Induced Massive Collagen Deposits was Less in DPP4-deficient Rats

**Figure 4:**
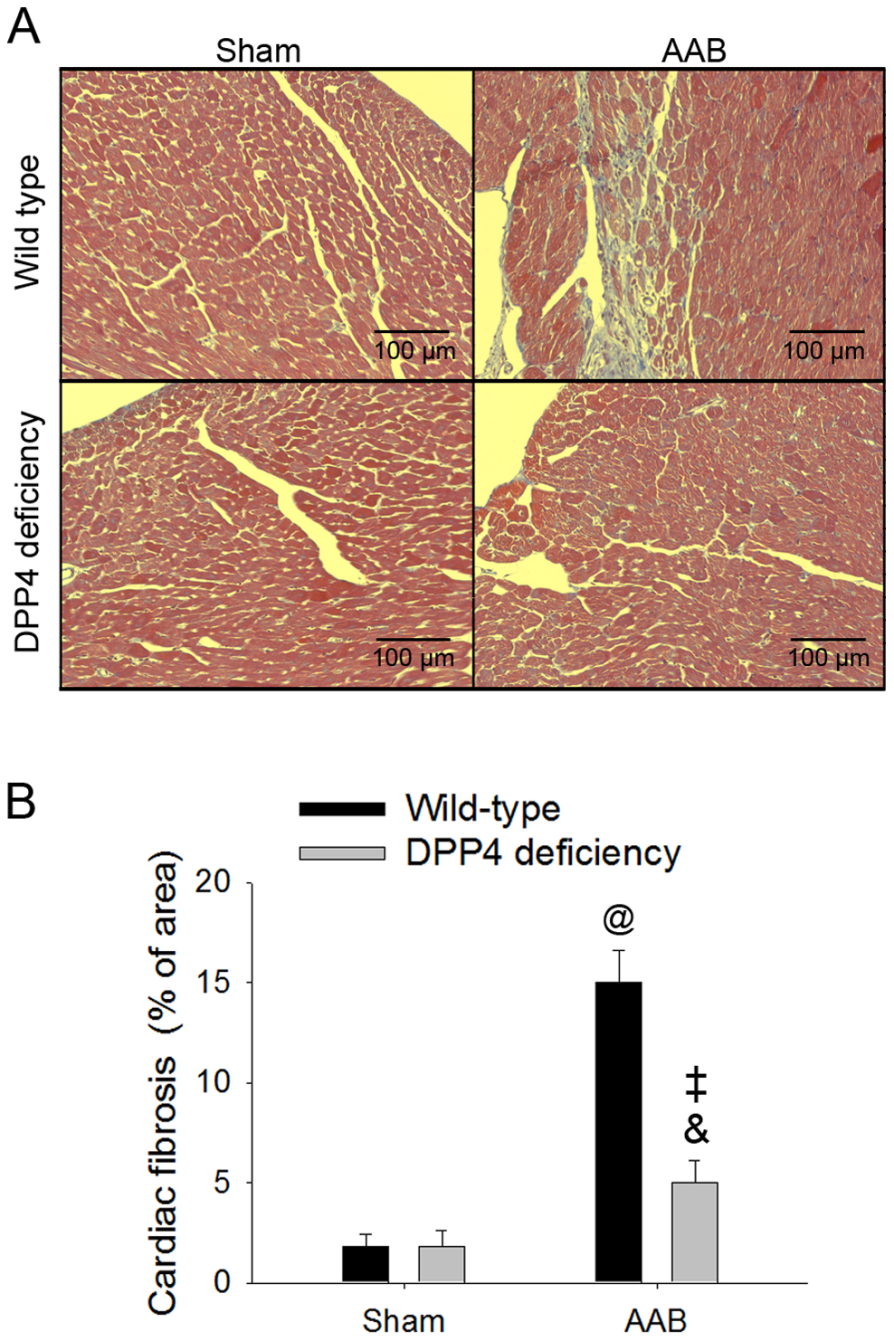
Effects of AAB on cardiac fibrosis. Masson’s trichrome staining revealed collagen deposits in blue, which was measured in wild-type and DPP4-deficient rats after 10 weeks of surgery. Representative masson’s trichrome sections (A), and quantification of the collagen accumulation (B). ^@^p<0.05 vs. wild-type sham, ^&^p<0.05 vs. wild-type AAB, ^‡^p<0.05 vs. DPP4 deficiency sham.

Masson’s trichrome staining revealed fibrosis in blue (Fig. 4A), and the percentage of fibrosis was also quantified (Fig. 4B). Collagen deposits was not found in myocardium at 6 weeks after AAB surgery (data not shown), but prominent collagen deposits appeared in myocardium of wild-type rats at 10 weeks after AAB surgery. The collagen deposit was less in DPP4-deficient rats.

## Discussion

Sustained pressure overload stimulates pathological cardiac hypertrophy, which changes calcium sensitivity and associates with the consequent defects in calcium homeostatsis [29]. Exitation-contraction coupling is a physiological process of converting an electrical stimulus to a mechanical response, which involves calcium release and uptake in association with the contraction and relaxation of heart [30]. The alteration of calcium handling related genes may result in heart failure [30], [31]. These changes include the decrease of the sarcoplasmic reticulum calcium pump (SERCA) activity during heart failure [31]. SERCA is inhibited by phospholamban. When phospholamban is phosphorylated, its ability to inhibit SERCA is lost [32]. Gene transfer of antisense phospholamban can improve the contractile function in failing human myocardium [33]. The modulation of calcium handling is a strategy to improve function of falling heart. In our study, aging decreased the rate of relaxation in wild-type rat hearts, indicating an impairment of calcium reuptake. In chronic pressure overload model, the decreased of ventricular systolic and diastolic function were found, suggesting a deterioration of calcium homeostasis. DPP4-deficient rats had better cardiac performance than wild-type rats in response to aging and pressure overload. These results coincide with other studies. DPP4 inhibitor exhibited significant attenuation of heart failure related dysfunction and remarkably mitigated pulmonary congestion [34], [35]. Indeed, sepsis induced decrease of phospholamban phosphorylation level was alleviated by DPP4 deficiency, and associated with the cardiac function preservation [14]. The capability of regulating calcium homeostasis by DPP4 deficiency may be partly contributed to the cardiac function improvement during heart failure.

Hypertrophic cardiomyopathy is an example of myocardial energy depletion [36]. Change of fatty acid to glucose utilization is increased early in heart failure. The switch in substrate utilization is a protective mechanism, which allows the heart to produce more ATP per molecule of oxygen consumed [1], [37]. In advanced hypertrophy heart failure, insulin resistance develops in heart failure result in decreasing glucose metabolism, which reduces the overall ability of the heart to generate sufficient ATP [1], [36]. A strategy for metabolic intervention in heart failure is to modulate substrate utilization [36]. Cardiac specific overexpression of GLUT1 prevents the development of heart failure in pressure overload mice [38]. Increase of myocardial glucose uptake contributes to the improvement of left ventricular performance in dogs with pace-inducing dilated cardiomyopathy [39]. In our previous study, the cardiac GLUT4 expression and glucose uptake elevated in normal DPP4-difficient rats [15], [21], and remain higher than that in wild-type rats during stress [21]. The improvement of cardiac function during pressure overload in DPP4-deficient rats may be partly attributed to the modulation of energy utilization.

Lack of DPP4 activity resulted in accumulation of plasma GLP-1 in DPP4-deficient rats. Evidence from several studies suggested that GLP-1 receptor agonist improved cardiac function in heart failure in both animal and clinical studies [39]–[42]. GLP-1 or its receptor agonist improved glucose uptake in heart by increasing GLUT4 translocation, and led to cardiac performance preservation in dilated cardiomyopathy or myocardial infarction induced failing heart [16], [43], [44]. Besides glucose homeostasis controlling, GLP-1 increased AKT, ERK, and eNOS signaling to activate survival pathway, which were associated with the outcome improvement after myocardial infarction [13], [41]. Liraglutide, a GLP-1 receptor agonist, ameliorated high fat diet induced cardiac endoplasmic reticulum stress, lipid accumulation, collagen deposition, and preserved cardiac function via an AMPK dependent pathway [45]. In our study, GLP-1 concentration increased after AAB in DPP4-deficient rats but not in wild-type rats, while DPP4 activity increased only in wild-type rats. Similar result was found in clinical study, enhanced serum DPP4 activity has been observed in patients with cardiovascular disease [34], [46]. The plasma DPP4 activity was correlated negatively with cardiac function in heart failure patients [34]. Higher DPP4 activity involve in degradation of GLP-1, so the elevation of GLP-1 concentration was only found in DPP4-deficient rats. GLP-1 is mainly secreted by L cells in distal small intestine, and also secreted by microglial cell in areas of the brain distinct from the brainstem and the NTS neurons [47]. However, the precise mechanism that regulating GLP-1 secretion during pressure overload remains unclear. We speculated that a great quantity of GLP-1 accumulation during pressure overload in DPP4-deficient rats can lead to cardioprotection.

Ventricular hypertrophy is associated with cell death. Several factors implicated as triggers of cardiomyocyte apoptosis, including a great quantity cytosolic calcium concentration, the formation of oxygen free radicals, excess levels of Ang II and cytokines [48]. Cell number in ventricle after abdominal aortic banding was less in wild-type rats than that in DPP4-deficient rats. DPP4 deficiency increased the capability against stress in cardiomyocyte during abdominal aortic banding. In our previous study, DPP4-deficient cardiomyocytes preserved catalase activity after H_2_O_2_ exposure. Bax/Bcl2 ratio and caspase3 activity were also found to be lower in DPP4-deficient cardiomyocytes than in wild-type cardiomyocytes after exposure to H_2_O_2_. The improvement of cell viability during ROS stress was partly contributed to GLP-1 accumulation in DPP4-deficient cardiomyocytes. [21]. Progression of cell death result in the replacement of fibrosis in the ventricle is the major determinant of increased myocardial stiffness and myocardial dysfunction [49]. Ang II promote the development of myocardial fibrosis in hypertensive heart disease and chronic heart failure [50]. Administration of Ang II in cardiac fibroblasts stimulate cell proliferation and collagen synthesis [51], while suppression of Ang II signaling is a well-known treatment for cardiovascular diseases. GLP-1 was reported to decrease the circulating concentration of Ang II in healthy men [52], and also had an anti-hypertensive effect through the attenuation of Ang II -induced high-salt sensitivity [53]. In our study, whether higher GLP-1 level contribute to the lower Ang II concentration in DPP4-deficient rat, or cardiac function improvement lead to the decrease of Ang II secretion after ABB remain unclear. In addition, DPP4 inhibitor decreased cardiac mRNA expression of fibrosis markers, such as TGF-β1, TIMP-1, collagen type I α1 and III α1 in uremic rats [54], and was also shown to alleviate collagen deposits in ventricle during heart failure [34], [35]. It can be conceivable that DPP4 deficiency alleviated left ventricular fibrosis after AAB in rat, which associated with the amelioration of heart dysfunction.

Besides GLP-1, Stromal cell derived factor-1α (SDF-1α), another DPP4 substrate, was also shown to exert cardioprotective effect [7]. G-CSF administration in combination with DPP4 inhibitor leads to the stabilization of active SDF-1α, which attracted stem cells to the injury sites and improved outcome after myocardial infarction [55]. In addition to the peptidase activity, DPP4 involve in immune system. DPP4 known as CD26 is a homodimeric type II transmembrane glycoprotein [7], [56], which is one of the accessory molecules of helper T cells, mediates as a co-stimulatory effect on T cell activation [7]. In this study, the cardioprotective effect of DPP4 deficiency during heart failure may be involved in more than GLP-1 signaling.

In conclusion, we have demonstrated that DPP4-deficient rats had better cardiac performance than wild-type rats after AAB surgery, which was associated with an alleviation of Ang II level and collagen deposits. The cardioprotective effect of DPP4 deficiency may be partly contributed to a great quantity of GLP-1 accumulation. These results prove that DPP4 deficiency improved cardiovascular outcome after pressure overload. The safety of long term loss of DPP4 activity should be further concerned.
